# Generation of an Etiology Index Based on Confirmed Tissue Diagnosis Data to Assess Endemic Swine Etiology Activity in the United States of America

**DOI:** 10.1155/tbed/9910689

**Published:** 2026-06-09

**Authors:** Guilherme A. Cezar, Danyang Zhang, Rodger G. Main, Eric R. Burrough, Rafael R. Nicolino, Maria Rodrigues da Costa, Gustavo Silva, Daniel C. L. Linhares, Giovani Trevisan

**Affiliations:** ^1^ Department of Veterinary Diagnostic and Production Animal Medicine, Iowa State University, Ames, Iowa, USA, iastate.edu; ^2^ Department of Preventive Veterinary Medicine, School of Veterinary Medicine, Federal University of Minas Gerais, Belo Horizonte, Minas Gerais, Brazil, ufmg.br; ^3^ Centre for Epidemiology and Planetary Health, School of Veterinary Medicine and Biosciences, SRUC (Scotland’s Rural College), Edinburgh, UK

**Keywords:** bootstrap, disease, EARS, matrix, monitoring, pathology, score

## Abstract

Global pork production has increased substantially over the past few decades, making swine a critical source of animal protein. However, endemic diseases in pigs continue to pose significant challenges to animal health, productivity, and food security. Several etiologies affect farm‐level performance and have broader implications for zoonotic risks and public health. Many diagnostic cases are submitted to veterinary diagnostic laboratories, and their aggregation can yield insights into etiological activity. Therefore, this study aimed to develop a composite etiology index using confirmed tissue diagnosis data from the Iowa State University Veterinary Diagnostic Laboratory (ISU‐VDL) to assess endemic etiology activity across the United States. A total of 59,950 porcine tissue cases from 2020 to 2024 were analyzed, focusing on 81 etiologies of bacterial, viral, parasitic, and metabolic/intoxication origin. Four normalized variables: disease occurrence, codiagnosis, state occurrence, and Early Aberration Reporting System (EARS) alarms were integrated using the CompidexR package to generate a weighted index ranging from 0.01 to 1. Temporal consistency was evaluated using the Manhattan distance, Spearman’s correlation, and the Wilcoxon signed‐rank test. Also, a bootstrap resampling method was developed to detect anomalies in the distribution of etiologies. The index demonstrated strong year‐over‐year stability, with porcine reproductive and respiratory syndrome virus (PRRSV) and *Streptococcus suis* consistently receiving yearly highest scores. Reemerging viruses like porcine sapovirus (PSaV) and astrovirus (AsV) showed notable increases in index values, reflecting rising diagnostic activity and geographic spread. Bootstrap analysis showed that over 55% of etiologies fell within expected confidence intervals (CIs) and had low root mean square errors (RMSEs), detecting anomalies such as PCV2 occurrences in 2024. The index summaries were visualized through an interactive Power BI dashboard, enabling dynamic exploration of etiology trends. This framework offers a scalable, reproducible tool for monitoring endemic swine diseases using routine diagnostic data. The ability to generate information on endemic etiology rankings can support decision‐makers with evidence‐based disease management and control. The developed model has flexibility and can be adapted to other species and disease systems. The index provided a robust foundation for enhancing surveillance of endemic pathogens in swine populations.

## 1. Introduction

In recent decades, global pork production has grown significantly, positioning it as one of the leading sources of animal‐derived proteins worldwide [1]. However, infectious diseases in swine present a significant challenge, threatening farm productivity and leading to broader consequences such as trade restrictions, food insecurity in low‐income countries, and risks of zoonotic transmission, thereby affecting public health and wildlife conservation [2]. Transboundary and foreign animal diseases can threaten different regions or countries, but endemic diseases cause constant animal threats and burdens [3].

The swine industry is diverse and multifaceted, encompassing a spectrum of operations from large‐scale, industrialized systems to smallholder farms and niche markets like show pigs [4]. Therefore, due to the complex structure of the swine industry, monitoring disease activity at the national level is a challenge. The Swine Disease Reporting System (SDRS) is an example of monitoring swine pathogens through diagnostic data, mainly polymerase chain reaction (PCR) or reverse transcriptase RT‐PCR assays [5] and sequencing data [6]. The project also includes confirmed tissue diagnosis data (tissue submissions analyzed by diagnosticians) from the Iowa State University Veterinary Diagnostic Laboratory (ISU‐VDL), which monitors the counts of diagnoses for several swine diseases [7].

However, to effectively guide control strategies, surveillance, and funding allocation, it is vital to determine which pathogens should be prioritized based on the monitored data, accounting for the complex industry network [8]. Disease prioritization is inherently complex, requiring well‐defined criteria [9] such as qualitative judgments or structured methodologies demonstrated in the World Health Organization (WHO) framework for identifying emerging infectious diseases needing urgent research [10]. Additionally, such criteria are often subjective and vary among stakeholders, with interdependencies complicating independent evaluations [11]. For instance, the 2013 U.S. outbreak of porcine epidemic diarrhea virus (PEDV) illustrates how emerging diseases can have both social and economic repercussions [12, 13]. However, once the pathogen becomes endemic in the country, demonstrating constant detections in several regions, it is essential to have additional information to determine if it is still a significant threat to animal health [14]. Therefore, there is a need to create automated infrastructures capable of monitoring and ranking disease/pathogen activity within a geographical region.

At the ISU‐VDL, disease diagnosis (Dx) codes were created to help laboratory personnel consistently document lesions and etiologies in a format amenable to efficient analysis and retrieval of aggregated disease diagnostic results [15]. These codes capture key information, including affected body systems, insult type, lesion type, and etiology/disease. The diagnostic process begins when clients submit samples and associated clinical histories [16]. Based on this information, diagnosticians use histopathology data along with selected ancillary tests (e.g., bacteriology, PCR, and sequencing) to establish diagnoses for each case. The resulting diagnoses are recorded in case reports and encoded using Dx codes [15]. Over the past decade, the ISU‐VDL has compiled over 100,000 porcine cases with confirmed tissue‐based diagnoses and more than 300 etiologies. This rich dataset offers valuable insights into swine disease trends and can inform epidemiological analyses [7].

The etiology index provides a robust and meaningful representation of pathogen activity based on diagnostic data by integrating multiple dimensions of information, such as case frequency, codetections, geographic distribution, and surveillance signals. Rather than merely proposing an index, this approach demonstrates the practical utility and relevance of synthesizing diverse data streams into a single, interpretable metric. The new comprehensive framework supports decision‐making and targeted interventions. Also, an automated infrastructure to gather and process the disease tissue diagnosis cases from veterinary diagnostic laboratories, updating the created indexes with regular frequency, could facilitate the accuracy of identifying changes in etiology activity. This study aimed to generate an automated etiology index based on confirmed tissue diagnosis data to assess endemic etiology activity based on samples submitted to the ISU‐VDL from 2020 to 2024.

## 2. Materials and Methods

### 2.1. Data Source

Historical etiology diagnosis data from porcine tissue cases received at the ISU‐VDL for diagnostic evaluation, starting from January 1^st^, 2020, to December 31^st^, 2024, were retrieved from the ISU‐VDL database and included in the analysis. The data was structured at the submission level using a SAS script (SAS Version 9.4, SAS Institute, Inc., Cary, NC), with each case submission identified by its accession ID. Additionally, ISU‐VDL retrieved case metadata comprised the received date, site state, farm type, and assigned Dx code(s). All producers and client information were anonymized before sharing by the ISU‐VDL to protect privacy, retaining only the site state (state‐level) location data needed to generate the “state occurrence” variable. In the data cleaning procedure, only etiologies related to viral, bacterial, parasitic, or metabolic/intoxication disorders were included in the analysis since the index was created to account for known endemic pathogens/etiologies. Etiologies caused by traumas such as “ear necrosis,” “torsion,” and “fibrosis” were removed from the analysis. Diagnoses classified as “not specified” or “ancillary” etiologies were also removed from the analysis. In addition, diagnostic codes about specific diseases with a common etiology cause were standardized for a single etiology name, e.g., “*E. coli* – hemolytic” and “*E. coli* – edema disease” were standardized as *Escherichia coli*.

### 2.2. Etiology Index Variables

The collated and cleaned data were then organized for variable creation utilized for the disease composition, summarizing the information at the etiology/disease level by year. The variables were created in R software (R Core Team 2021, Vienna, Austria), and algorithms were developed to compute the variables used in the index, standardizing them by dividing all the numbers by the maximum and minimum results obtained among the years within each variable using the described formulas below. This approach adjusts for differences in magnitude and scales all values to a standard range between 0 and 1, including the temporal differences along the years evaluated. The subscript *i* in the formulas denoted the specific disease or etiology, indicating that the formula was applied for each etiology in the dataset, comparing to the minimum and maximum values among all the etiologies throughout the years. Therefore, the etiology index was constructed independently within each year to capture the annual distribution, but its standardized values were calculated by comparing across all years in the dataset. Specifically, the maximum and minimum scores used for scaling are derived from the entire temporal span rather than a single year, allowing the index to highlight shifts in etiologies over time while maintaining consistent measurement across years, as described in the variables below.

#### 2.2.1. Disease Occurrence

Disease occurrence represents the total number of diagnostic cases attributed to each swine disease or etiology within a calendar year. Diagnoses were aggregated at the submission level, capturing the annual count of submission events of tissue cases with a confirmed diagnosis for each etiology across the dataset. The normalization formula applied was

Disease Occurrencei=xi−minximaxxi−minxi,

*x*_*i*_ = the number of diagnostic cases attributed to disease *i* in a given year.

#### 2.2.2. Disease Codiagnosis

Disease codiagnosis captures the extent to which a given swine disease or etiology was diagnosed and assigned in conjunction with other etiologies/diseases within a diagnostic submission. Specifically, it reflects the number of unique codiagnosed etiologies associated with each disease across all submissions (identified by the accession ID) within a calendar year. As an example, *Actinobacillus pleuropneumoniae* was codiagnosed with 17 different diseases/etiologies in 2024. This metric provides insights into potential coinfection relationships. For each etiology, the number of distinct codiagnosed etiologies was counted annually. The same normalization was applied as follows:

Co-diagnosisi=xi−minximaxxi−minxi,

*x*_*i*_ = the number of distinct codiagnosed etiologies associated with disease *i* across all diagnostic submissions in a given year.

#### 2.2.3. Number of Early Aberration Reporting System (EARS) Alarms

EARS alarms quantify the frequency of aberrant diagnostic activity for each swine disease etiology, as detected by syndromic surveillance. Specifically, the EARS C1 model was applied to weekly diagnosis counts for each etiology, following the methodology previously described[7, 17]. Based on the preceding 7 weeks of data, the model flags a weekly count as an alarm if it exceeds the expected baseline by more than three standard deviations. The total number of alarms triggered within a calendar year was summed for each etiology to produce the annual EARS alarm counts. This metric reflects unusual or potentially outbreak‐related diagnostic patterns. The normalization formula applied was

EARS alarmsi=xi−minximaxxi−minxi,

*x*_*i*_ = the total number of EARS C1 alarms triggered for etiology *i* within a calendar year.

#### 2.2.4. State Occurrence

State occurrence variable was derived from the “site state” information in the diagnostic database and represents the geographic distribution of each diagnosed swine disease/etiology across the United States of America. Specifically, it quantifies the number of distinct states where a given etiology was diagnosed within a calendar year. The metric serves as a proxy for each disease’s regional spread. The previous methodology was applied in the normalized formula

State Occurrencei= xi−minximaxxi−minxi,

*x*_*i*_ = the number of distinct U.S. states where etiology *i* was diagnosed within a calendar year.

Then, the R package CompidexR [18] function “calc_index” was utilized to create the final index. The function used the average method “simple” and the scaling method “min–max," which performed a multistep process: (1) computed the standardized ranged variables after normalization using min–max calculation; (2) calculated sensitivity indices (Si) to evaluate the influence of each variable on the final index; (3) assessed multicollinearity through the Variance Inflation Factor (VIF) to identify highly collinear variables; and (4) adjusted and optimized variables weight based on the Si and VIF, considering the final index must sum to 1 and cannot be negative. These indices quantify how changes in individual variables affect the composite score, providing insights into the robustness and responsiveness of the index structure. After the weight was attributed, the final index formula was implemented by adding the corresponding weight of each variable, making the index range from a minimum of 0.01–1 as the maximum values a disease/etiology can achieve.

Final Index =∑wi⋅xi′,

w_i_: The weight assigned to the *i* ^th^ variable, reflecting its relative importance in the index.

x_i_′: The normalized value of the *i* ^th^ variable, scaled to a standard range using min–max normalization.

### 2.3. Assessment of Temporal Changes in Disease/Etiology Over Time

As part of the process for demonstrating consistency for the composite etiology index, a Manhattan distance (distance‐based model) was implemented to assess temporal changes in the disease/etiology rank. Traditionally used to measure spatial proximity [19], the models were adapted to quantify statistical distances between disease ranks based on their index value across consecutive years. The Manhattan distance compared disease rankings derived from index values between successive years (2020–2021, 2021–2022, 2022–2023, and 2023–2024), allowing for the quantification of year‐over‐year shifts. The Manhattan distance was computed using the following formula:

DManhattan=1n∑i=1n Rit+1−Rit,

where *R*_*i*_^(*t*)^ and *R*_*i*_^(*t* + 1)^ represented the ranks of etiology *i* in year *t* and *t + 1*, respectively, and *n* is the total number of diseases evaluated. This calculation was performed using R software, where diseases were ranked based on their composite index values, and the mean absolute difference in ranks was computed. A subset of data analysis was used to explore further significant shifts in the top 10 diseases (based on the rank in the second year of each pair). A scatter plot was generated to visualize rank changes, with a reference line indicating perfect rank stability and disease labels added for interpretability. The Wilcoxon signed‐rank test was also applied to compare paired ranks, testing for statistically significant differences (*p*‐value < 0.05) in the ranks of etiology indexes over time. In addition, a Spearman correlation analysis was used to assess the correlation between index values across years.

### 2.4. Bootstrap Model as a Tool to Detect Etiology Anomalies

A bootstrap resampling procedure was conducted in R software using diagnostic data from 2020 to 2024 to detect variability and possible anomalies by creating a bootstrap score as continuous data. Five hundred bootstrap iterations were performed, each initiated by randomly sampling the unique case identifiers (accession IDs) from the original dataset with replacement. The frequency of each sampled identifier was calculated, and the dataset was reconstructed accordingly using row replication to preserve the structure of individual diagnostic submissions. For each resampled dataset, three normalized metrics were recalculated: “disease occurrence,” “disease codiagnosis,” and “state occurrence.” These metrics were normalized using the same min–max scaling method described previously. The resulting variables were merged, the missing values were imputed with zeros, and a bootstrap score was computed by summing the normalized components.

This process was repeated across all bootstrap samples, and for each disease etiology, the mean score value and its 2.5^th^ and 97.5^th^ percentile bounds were calculated to form a 95% confidence interval (CI). The bootstrap‐based index was assessed on a scale ranging from 0 to 3, reflecting the exclusion of the EARS alarm variable, whose sampling distribution is not suitable for obtaining by using the bootstrap. Bootstrap scores calculated from each year were compared with the following year, as described in the Manhattan distance model, and compared against the bootstrap CIs to validate the performance of the bootstrap‐derived index. The proportion of values falling within the bootstrap intervals and the root mean square error (RMSE) between the predicted and observed values were computed to evaluate predictive consistency.

### 2.5. Etiology Index Visualization

The final dataset, which included all calculated variables and the composite index for each disease etiology by year, was exported from R as comma‐separated value (CSV) files. Then, the final file was imported into a business intelligence analytical tool, Power BI Desktop (Microsoft, Redmond, WA), to facilitate interactive visualization of temporal trends and shifts in the index. Using Power BI’s built‐in tools, dynamic dashboards were created to allow users to filter and explore the data by year and etiology. A generated chart with index results over the years was converted into a dashboard and subsequently published to Power BI Pro (Microsoft, Redmond, WA) and made available through the link (https://fieldepi.org/sdrs/dashboards/, *Disease Index Dashboard*). The underlying algorithms were configured to recalculate the composite index upon each data refresh to ensure that the dashboard remained up to date, reflecting newly added diagnostic cases.

## 3. Results

### 3.1. Data Source and Variables Created for the Final Index

A total of 59,950 cases were utilized to create the etiology index, and 81 diseases/etiologies from bacterial, viral, metabolic/intoxication disorders, and parasitic insults were incorporated after the data cleaning and collation. A total of 167 etiologies not related to the insult categories utilized for this analysis were removed from the database. The final etiology index goes from 0.01 to 1, where 1 is the highest possible score for one etiology. The weights assigned for each variable were disease occurrence (0.50), number of EARS alarms (0.26), state occurrence (0.15), and disease codiagnosis (0.09). Disease codiagnosis had the lowest weight due to its high VIF (10.05) and Si, demonstrating its high correlation with the other three variables and impact on the final index. These results explain the disease codiagnosis variable, lowest weight. Following the same rationale, the variables with the highest to lowest VIF were state occurrence (VIF = 8.10; *Si* = 0.27), number of EARS alarms (VIF = 2.69; *Si* = 0.24), and disease occurrence (VIF = 2.62; *Si* = 0.19). After the weight implementation, the index was calculated per year for all diseases/etiologies. As an example, the results of the 10 diseases/etiologies receiving the highest index in 2024 are displayed in Table 1.

**Table 1:** The 10 diseases/etiologies receiving the highest indexes in 2024 after multiplying each variable’s results by its respective weight: disease occurrence (0.50), number of EARS alarms (0.26), state occurrence (0.15), and disease codiagnosis (0.09).

Etiology	Disease occurrence	Disease codiagnosis	EARS alarms	State occurrence	Final index
PRRSV	0.45	0.08	0.26	0.14	0.93
*Streptococcus suis*	0.32	0.08	0.26	0.14	0.80
*Pasteurella multocida*	0.13	0.06	0.26	0.11	0.56
Influenza A virus	0.17	0.07	0.21	0.10	0.55
Rotavirus	0.12	0.05	0.16	0.11	0.44
*Glaesserella parasuis*	0.14	0.06	0.13	0.10	0.43
*Escherichia coli*	0.11	0.06	0.13	0.11	0.41
*Salmonella enterica*	0.07	0.05	0.18	0.09	0.39
PCV2	0.07	0.05	0.16	0.09	0.37
*Mycoplasma hyopneumoniae*	0.04	0.04	0.18	0.10	0.36

Abbreviations: PCV2, porcine circovirus 2; PRRSV, porcine reproductive and respiratory syndrome virus.

### 3.2. Assessment of Temporal Changes in Disease/Etiology Over Time

The etiology index demonstrated strong consistency across years. The Manhattan distance analysis was applied to the rank of diseases based on the index and showed a mean rank change of six positions across all years. However, within the 10 diseases/etiologies receiving the highest indexes, the mean change was three positions, indicating that the top‐ranked diseases have high stability by changing their ranks less than the analysis including all the etiologies/pathogens. In the assessment year‐to‐year, the Wilcoxon signed‐rank test was not statistically significant for mean rank changes, which also showed that the disease rank didn’t have significant changes over the years, considering all the diseases/etiologies. A mean Spearman correlation coefficient across the years of 0.92 among the index values also supported a strong consistency in the index year‐over‐year (Table 2).

**Table 2:** The Manhattan distance model, the Wilcoxon signed‐rank test, and Spearman correlation results year‐to‐year.

Years	Mean rank change	Mean rank change (top 10 etiologies)	Wilcoxon signed‐rank test (*p*‐value)	Spearman correlation of index values
2020–2021	5	1	0.8307	0.95
2021–2022	7	3	0.5887	0.89
2022–2023	6	4	0.5069	0.91
2023–2024	6	4	0.9428	0.93

**Figure 1:**
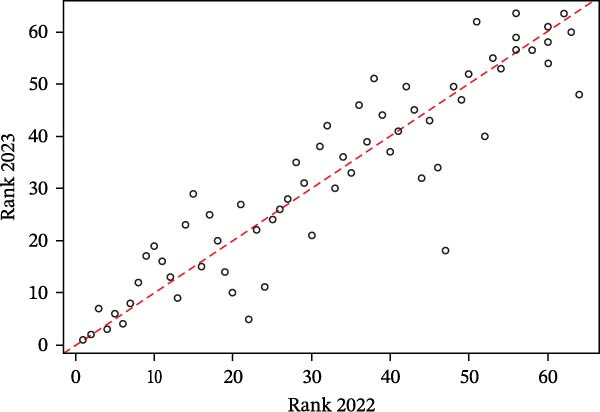
A scatter plot to visualize rank changes comparing 2022 and 2023 etiologies. *X*‐axis rank of etiology in 2022 and *Y*‐axis rank of etiologies in 2023. A reference diagonal dotted red line indicates perfect rank agreement.

A different pattern of disease stability was also identified through the scatter plots. It was visible that the extremes of the plots (highest and lowest ranks) had more stability throughout the years, whereas the highest variations started after the top 10 ranked etiologies. However, as the mean distance analysis has shown, there was not much variability in the ranks among the 1‐year‐olds compared with the others, as represented in Figure 1, where the majority of the etiologies are near the red line, representing a minor rank change.

**Figure 2:**
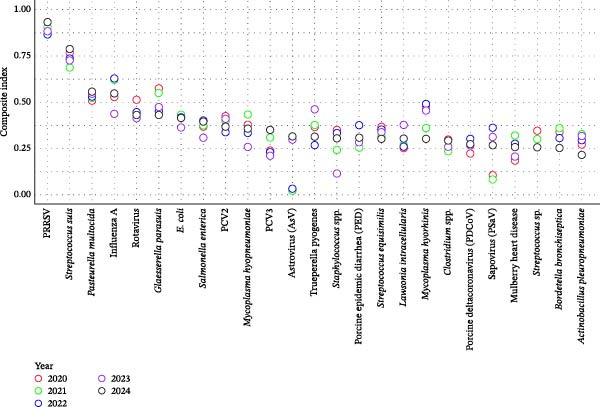
The 25 etiologies receiving the highest indexes in 2024 and their variation over the years. Each color dot represents a year: 2020 (red), 2021 (green), 2022 (blue), 2023 (purple), and 2024 (black). *E. coli, Escherichia coli*; PCV2, porcine circovirus 2; PCV3, porcine circovirus 3; PRRSV, porcine reproductive and respiratory syndrome virus.

Among the diseases/etiologies receiving the highest indexes, porcine reproductive and respiratory syndrome virus (PRRSV) and *Streptococcus suis* ranked first and second consistently across the years, with indexes always equal to or above 0.70 (Figure 2). *Glaesserella parasuis*, *Pasteurella multocida*, influenza A virus, and rotavirus were the other etiologies remaining in the top 10 ranked etiologies since 2020, varying among ranks 3 and 8. In addition, porcine circovirus 2 (PCV2) and *E. coli* stayed inside the top 10 ranked etiologies in 4 out of 5 years, demonstrating stability within the top‐ranked etiologies. Other diseases/etiologies that at some points were inside the top 10 ranked were *Mycoplasma hyorhinis* (2021, 2022, and 2023); *Mycoplasma hyopneumoniae* (2020, 2021, and 2024); *Salmonella enterica* (2022 and 2024); *Trueperella pyogenes* (2023 and 2021); coccidiosis (2023); and PEDV (2022).

When expanding the analysis for the top 25 etiologies, porcine sapovirus (PSaV) and astrovirus (AsV) had the highest rise in their indexes compared with any other etiologies in the database. As reemerging viruses in the United States [20, 21], the number of diagnosed cases of these viruses increased substantially, which contributed to the index increase. PSaV jumped from 38 cases, 0 alarms generated by the EARS model, and diagnosed in 6 states in 2021 (final index = 0.08) to 177 cases and 8 alarms detected for an abnormal weekly count of cases and diagnosed in 19 states, more than quadrupling its final index in 2022 (0.36). A similar pattern happened with AsV, where it jumped from 10 cases in 2022, diagnosed in 4 states (final index = 0.03), to 102 cases in 2023, diagnosed in 14 different states, reaching the highest index in 2024 (0.32), where 127 cases were associated with 26 different etiologies, diagnosed in 16 states, and 7 alarms on the EARS model.

Still in the top 20 etiologies, two enteric coronaviruses were also present: PEDV and porcine delta‐coronavirus (PDCoV). Since they emerged in the U.S. swine industry in 2013, they have caused severe losses [12] but since then, they have become endemic in the country, being frequently detected by RT‐PCR in diagnostic submissions to veterinary diagnostic laboratories [22]. In the timeline of this analysis (2020–2024), both PEDV and PDCoV reached their highest indexes in 2022, 0.38 and 0.30, respectively.

On the other hand, metabolic/intoxication disorders were ranked as the disease/etiology with the lowest indexes. Copper, calcium, zinc, iron, and selenium intoxication have 10 or fewer cases across the years, like some vitamin deficiencies, such as vitamin A or vitamin D. Similar for mycotoxins such as zearalenone, aflatoxins, and fumonisins that had three or fewer confirmed tissue diagnoses per year. The low number of cases also contributes to high rank changes in the disease/etiologies with low indexes since a minor increase in the number of cases from 1 year to another can result in a larger rank change. As an example, from 2021 to 2022, it decreased by 12 positions due to a change from 9 to 2 total number of cases.

### 3.3. Bootstrap Model as a Tool to Detect Etiology Anomalies

The bootstrap analysis used the same systematic approach as the Manhattan distance analysis, using the previous year to calculate a range of plausible bootstrap etiology index values for each etiology in the next predicted year. All the years demonstrated a within‐confidence‐interval rate higher than 55% and a low RMSE (Table 3).

**Table 3:** Bootstrap analysis results, including overall and top 10 etiologies proportions within the predicted confidence interval (CI) for bootstrap score and overall root mean square error (RMSE) by year.

Years	Proportion of etiologies within the expected CI (%)	Proportion of etiologies within the expected CI (top 10 etiologies)	RMSE
2020–2021	55.22	50.00	0.066
2021–2022	62.90	70.00	0.086
2022–2023	68.75	70.00	0.072
2023–2024	71.42	90.00	0.062

**Figure 3:**
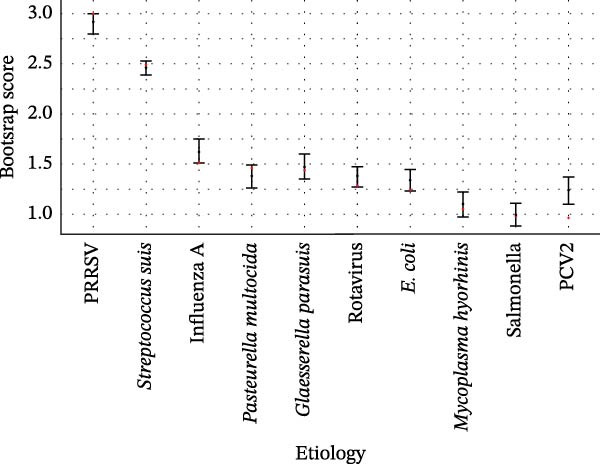
Top 10 etiologies in 2024 according to their observed bootstrap score (red dot) based on the bootstrap model and the 95% confidence interval prediction based on 2023 data. *E. coli, Escherichia coli*; PCV2, porcine circovirus 2; PRRSV, porcine reproductive and respiratory syndrome virus.

The RMSE results demonstrated a low difference between the predicted values by the bootstrap and observed values. Therefore, the results combined with the rates of percentage of etiologies within the CIs also supported the low variability of the bootstrap score among the etiologies/diseases throughout the years. In the top 10 etiologies, the proportion within the CI was even higher than the overall results, demonstrating more consistency in these top 10 etiologies, as emphasized by the Manhattan distance results. However, since the top 10 etiologies have high indexes, a change in the pattern of etiologies in a year can affect the prediction for the following year. Diseases/etiologies such as the influenza A virus and PRRSV that have a higher strain diversity [23, 24] and the occurrence of more or less aggressive strains can affect the index due to a major change in the variables. The comparison between 2020 and 2021 was an example where the bootstrap methodology identified for the first time PRRSV out of the predicted CI, and within these years, there was an emergence of a new highly pathogenic PRRSV variant, further identified as the lineage 1 C.5 strain ([25, 26]. The PRRSV example may indicate the capability of the bootstrap to identify anomalies in etiology patterns from 1 year to another. In the top 10 etiologies comparison between 2023 and 2024 using bootstrap scores, visualized in Figure 3, the plot identified a major change in the PCV2 observed value (0.14 below the lower limit of the 95% CI) compared with the overall RMSE.

It is essential to highlight that the RMSE results described in Table 3 can be visualized in Figure 3 as well, where, apart from PCV2, the observed value had a minimum distance from the predicted mean by the bootstrap. Therefore, the bootstrap score can be used to identify major changes in endemic etiologies based on historical diagnosis data, considering three out of four variables included in the final index. In the end, the bootstrap visualized possible etiologies that were going outside the predicted interval to alert the investigators in case an etiology was having an atypical behavior, as was seen for PCV2 in 2024 based on 2023 data.

### 3.4. Etiology Index Visualization

**Figure 4:**
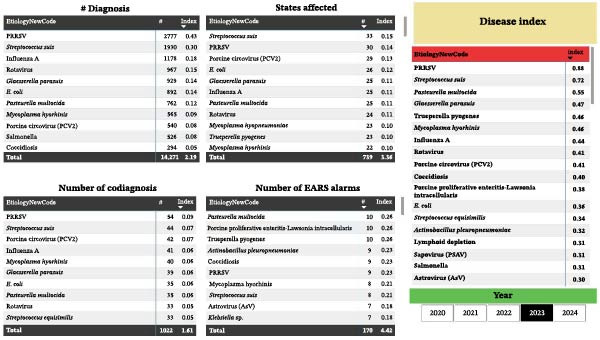
Dashboard to visualize the etiology index for the year 2023, including the absolute values by variable and their contribution portion for the final index for internal laboratory usage.

The final etiology index, with all variables contributing to the etiology index—disease occurrence, disease codiagnosis, EARS alarms, and state occurrence—was exported to a CSV file as the final output, with the absolute number for each variable and the corresponding index, and was connected and integrated into two Power BI dashboards. The final setup enabled updates by running the R code to incorporate new diagnostic data from the ongoing year. Two final dashboards were created in Power BI. Figure 4 shows the first dashboard, where each index variable is displayed in black tables, visualizing the absolute count and its contribution to the final score. Then, on the far right of Figure 4, the etiologies are ordered from the highest to lowest index. Furthermore, clicking the year button at the bottom right allows the user to view each year interactively. This dashboard is password‐protected for internal laboratory use as it contains confidential data.

**Figure 5:**
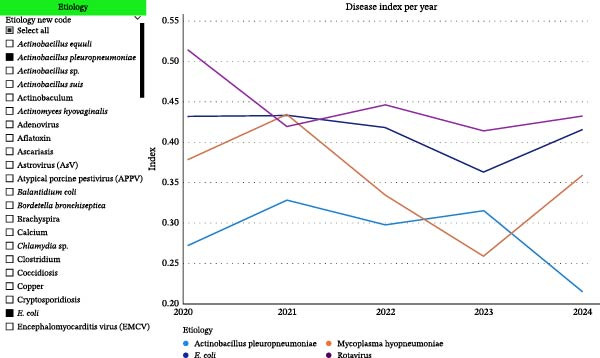
Dashboard to visualize the selected etiology index over time. By clicking on the etiologies in the table, the dashboard will display the line chart to visualize the indexes over time, facilitating the comparison among specific etiologies throughout the years.

In Figure 5, the second dashboard is displayed, allowing the user to compare etiology indexes over time by selecting the desired etiology. By selecting a specific etiology in the table of etiologies (left side of Figure 5), which contains all the etiologies in the index, the dashboard will automatically display a line chart showing the index values for this disease/etiology over the years. This dashboard was made publicly available via the link (https://fieldepi.org/sdrs/dashboards/, *Disease Index Dashboard*) and can be continuously updated by adding prospective data.

## 4. Discussion

This study presents a novel, data‐driven approach to monitoring endemic porcine etiologies in the United States of America by developing a composite etiology index based on confirmed tissue diagnoses from a central veterinary diagnostic laboratory. Integrating multiple epidemiological indicators—disease occurrence, geographic spread, codiagnosis patterns, and syndromic surveillance alarms—into a single, weighted index provides a robust framework for assessing etiology activity over time. The study developed a methodology that addressed the need for objective, scalable, and easy‐to‐interpret tools to rank endemic etiologies using diagnostic data.

The etiology index demonstrated strong temporal consistency, evidenced by high Spearman correlations and low Manhattan distances across years. This stability reinforces the reliability of the index as a longitudinal monitoring tool. Notably, the top‐ranked etiologies (i.e., PRRSV, *S. suis*, *Pasteurella multocida*, and influenza A virus) remained consistently among the most active etiologies, aligning with their known endemicity in U.S. swine production systems [27, 28]. For PRRSV and influenza A virus, the findings are consistent with previous studies highlighting the persistent detection of these etiologies by PCR detection [5, 29] and reports of prevalence in the U.S. swine herd [30, 31], impacting herd health. In addition, *S. suis* has been reported as a major bacterial etiology affecting pigs, responsible for economic losses in the global swine industry due to various clinical conditions in swine, such as pneumonia, septicemia, endocarditis, and meningitis [28, 32].

Including the EARS model as a syndromic surveillance component adds a valuable layer of sensitivity to detect aberrant diagnostic patterns. While the EARS variable contributed significantly to the final index (weight = 0.26), its exclusion from the bootstrap model, due to simulation constraints, did not compromise the overall predictive performance. The bootstrap model was an additional tool created for detecting anomalies using the same database, with over 55% of etiologies falling within the predicted CIs and low RMSE values across years. However, it is important to address that the bootstrap score did not include the EARS model data. Therefore, it is essential to highlight the limitations of the bootstrap methodology by not including all the variables that contributed to the etiology index. On the other hand, notably, the bootstrap framework served as a warning system, flagging atypical behavior in etiologies such as PCV2, which exhibited a marked decline in 2024 compared to prior years. The year of 2023 had an increased activity of PCV2 and PCV3 based on PCR diagnostic cases [33], in which PCV2 achieved a peak of 49% of positive cases coming from sow farms, pathogens in the Spring of 2023, and a larger percentage of tissue cases (38%), which might explain the prediction of the bootstrap of a high index in 2024 since 2023 had a higher PCV2 activity in the field.

The index also captured trends in reemerging viruses such as PSaV and AsV, which showed substantial increases in diagnostic activity and geographic spread. These findings underscore the utility of the index in detecting shifts in endemic etiology dynamics, which may otherwise go unnoticed in traditional surveillance systems. In 2020, a genetically divergent strain of PSaV emerged [34] and began to cause more diarrhea cases in piglets [20], which impacted the number of cases in the following years. Also, in 2023, cases with macroscopic lesions such as lobular atelectasis of a cranioventral lobe in lungs associated with positive PCR cases for porcine AsV 4 started to increase, demonstrating AsV as a pathogen that can cause respiratory clinical signs in swine [21], which also impacted the increased number of AsV in 2023. The ability to quantify and visualize such changes through an interactive Power BI dashboard enhances the practical applicability of this tool for veterinarians, producers, and policymakers.

Despite its strengths, the index has limitations. One notable constraint is its inability to capture foreign animal diseases, which are typically absent from routine diagnostic submissions and may only be detected through targeted surveillance or specialized reporting systems [35]. As a result, the index utility in monitoring exotic disease threats is not recommended since it will have a delayed response compared to syndromic surveillance systems. Additionally, the reliance on confirmed tissue diagnoses may underrepresent etiologies primarily detected through other diagnostic modalities (e.g., PCR on oral fluids or feces). This is the case of PEDV and PDCoV, where, even though postepidemic, the incidence has decreased in the United States [36], and the diagnosis usually relies on positive RT‐PCR cases associated with clinical signs, which can be highlighted by the still high number of submissions tested by RT‐PCR for the enteric coronaviruses (Trevisan et al., 2021c). Also, the index is sensitive to diagnostic submission patterns, which may be influenced by factors unrelated to true disease prevalence, such as producer awareness, sample submission logistics, or economic conditions [37].

Although the VIF results revealed multicollinearity among variables, codiagnosis (VIF = 10.05) and state occurrence (VIF = 8.10), we elected to retain these variables in the final index due to their epidemiological relevance. Codiagnosis captured the frequency with which etiologies interact with other etiologies, providing insights into coinfection dynamics that cannot be fully explained by case counts alone. Similarly, state occurrence reflects the geographic spread of an etiology. While both variables are naturally correlated with disease occurrence (i.e., etiologies with higher case counts might co‐occur more frequently and be detected across more states), excluding them would risk oversimplifying the multifactorial nature of etiology activity. The CompidexR framework addresses multicollinearity by penalizing the weights of highly correlated variables [18], thereby reducing their influence while preserving their interpretive value. The approach ensured that the final index remains biologically meaningful, balancing statistical rigor with epidemiological relevance.

Nevertheless, a key strength of the index lies in its ability to quantify disease and etiology diagnoses in a standardized format, offering a foundation for future models that could incorporate economic data and expert opinion to support decision‐making. While such integration is beyond the scope of this study, it represents a promising direction for future research. In addition, the framework can be easily adapted to other veterinary diagnostic laboratories, which may facilitate a future index including data from multiple laboratories. It is important to emphasize that the index created by the authors is appropriate for monitoring relative trends, ranking endemic etiologies, and identifying shifts in diagnostic activity over time rather than for estimating absolute prevalence or incidence at the national level. Readers should avoid overinterpreting the results as surveillance of all U.S. swine populations; instead, the index should be viewed as a reproducible tool for contextualizing laboratory‑based diagnostic data, supporting evidence‑based prioritization within the scope of the ISU‑VDL network.

In conclusion, this study provides a scalable, reproducible framework for monitoring endemic etiology diagnostics in swine populations. The framework can be customized to a variety of different applications for endemic etiology monitoring, as demonstrated in the manuscript using dashboards and bootstrap models. By leveraging routinely collected diagnostic data and statistical modeling, the etiology index offers a dynamic tool to support evidence‐based decision‐making in animal health. Its integration into a real‐time dashboard further enhances its utility for strategic planning in swine health management in a friendly format available at the website (https://fieldepi.org/sdrs/dashboards/, *Disease Index Dashboard*). As animal health challenges continue to increase, tools like this index can support animal health data‐driven decisions, and this framework has potential adaptability for monitoring endemic and reemerging etiologies of all livestock species.

## Funding

This project was funded by the Swine Health Information Center (SHIC) (Grant 24‐016) for the investigators, Drs. Giovani Trevisan and Daniel C. L. Linhares.

## Conflicts of Interest

The authors declare no conflicts of interest.

## Data Availability

Restrictions apply to access to additional data, and the standard operating procedure (SOP) that was used to aggregate the information was due to the client’s and VDLs’ confidentiality and is not publicly available. The SOP procedure can be made available upon reasonable request and approval by the SDRS principal investigators (Dr. Giovani Trevisan at trevisan@iastate.edu, (Dr. Daniel C. L. Linhares at linhares@iastate.edu) and the VDL director (Dr. Rodger G. Main at main@iastate.edu).
